# 2D crystal structure and anisotropic magnetism of GdAu_6.75−x_Al_0.5+x_ (x ≈ 0.54)

**DOI:** 10.1038/s41598-022-17068-4

**Published:** 2022-07-30

**Authors:** D. C. Joshi, G. H. Gebresenbut, A. Fischer, A. Rydh, U. Häussermann, P. Nordblad, R. Mathieu

**Affiliations:** 1 Department of Materials Science and Engineering, Uppsala University, Box 35, 751 03 Uppsala, Sweden; 2 Department of Chemistry-Ångström Laboratory, Uppsala University, 751 21 Uppsala, Sweden; 3 Institute of Physics, Augsburg University, 86159 Augsburg, Germany; 4 Department of Physics, Stockholm University, 106 91 Stockholm, Sweden; 5 Department of Materials and Environmental Chemistry, Stockholm University, 106 91 Stockholm, Sweden

**Keywords:** Magnetic properties and materials, Structure of solids and liquids

## Abstract

Exploration of the gold-rich part of the ternary Gd–Au–Al system afforded the intermetallic compound GdAu_6.75−x_Al_0.5+x_ (x ≈ 0.54) which was structurally characterized by single crystal X-ray diffraction (*Pnma*, a = 18.7847(4) Å, b = 23.8208(5) Å, c = 5.3010(1) Å). GdAu_6.75−x_Al_0.5+x_ crystallizes in a previously unknown structure type featuring layers of Gd_2_(Au, Al)_29_ and Gd_2_(Au, Al)_28_ clusters which are arranged as in a close-packing parallel to the *ac* plane. The Gd substructure corresponds to slightly corrugated 3^6^ nets (d_Gd–Gd_ = 5.30–5.41 Å) which are stacked on top of each other along the *b* direction with alternating short (5.4, 5.6 Å, within layers) and long distances (6.4 Å, between layers). The title compound has been discussed with respect to a quasicrystal approximant (1/1 AC) GdAu_5.3_Al in the same system. The magnetic properties of GdAu_6.75−x_Al_0.5+x_ were found to be reminiscent to those of some ternary ACs, with sharp peaks in the temperature dependent magnetization, and metamagnetic-like transitions. The material becomes antiferromagnetic below 25 K; magnetometry results suggest that the antiferromagnetic state is composed of ferromagnetic *ac* planes, coupled antiferromagnetically along the *b* direction.

## Introduction

Investigations of the rare-earth (RE) containing RE-Cd, RE-Cd–Mg and RE-Au–Al systems have been especially fruitful for studying magnetism in quasicrystals (QC)s and their related approximant crystals (AC)s^1–4^. These QCs and ACs are of Tsai-type and thus built of clusters consisting of four concentric shells and centered by a tetrahedral moiety. RE atoms are arranged into icosahedra and represent one of the cluster shells^5–8^.

Phase diagrams exhibiting Tsai-type QCs generally also contain the compositionally similar 1/1 AC phase. However, since the stability of Tsai-type QCs is linked to a very narrow valence electron per atom ratio, 1/1 ACs are found much more frequently than QCs^9^. This seems to be the case for the Gd–Au–Al system for which only the 1/1 AC phase has been reported. Yet the Gd–Au–Al AC phase displays an extraordinary broad range of composition, Gd_14_Au_x_Al_86−x_, x = 49–72, and intriguing Au–Al composition-driven magnetic property changes^10–12^.

Frequently QC phases adopt a slightly RE-poorer composition (~ 12 at.%) in intermetallic phase diagrams compared to their related 1/1 AC phase (~ 14 at.%), and are easily overlooked because of their comparatively low temperature of peritecitc decomposition (into AC phase and melt)^13^. Only careful studies of the liquidus for the RE-poor region (< 5 at. % RE) may reveal QC phases^13,14^. In the case of ternary phase diagrams. It is sometimes possible to apply a pseudo-binary approach (e.g. Gd_14_X_86_) in which the majority component X is replaced by a binary mixture at or close the eutectic composition^15^. This can provide a situation where the liquidus temperature is below the peritectic temperature of the QC phase and thus allow access to QC phase through crystallization from the melt. For the Au–Al (X) system the composition Au_0.82_Al_0.18_ is close to a deep eutectic point with melting temperature of 525 °C^16^.

Here we report the synthesis of a new ternary intermetallic compounds in the Gd–Au–Al system using this approach. The structure of this new Gd–Au–Al phase displays a peculiar 2D character. The phase orders antiferromagnetically below 25 K, as a result of the antiferromagnetic coupling of ferromagnetic *ac* planes along the *b* direction of the structure. Interestingly the new compound has a chemical composition which is very similar to QC and a phase relation with AC phases as in a binary phase diagram. We discuss the observed magnetic properties in the light of those of their structural crystallographic siblings.

## Results and discussion

### Partial Pseudo-binary Gd–(Au_0.82_Al_0.18_) system

**Figure 1:**
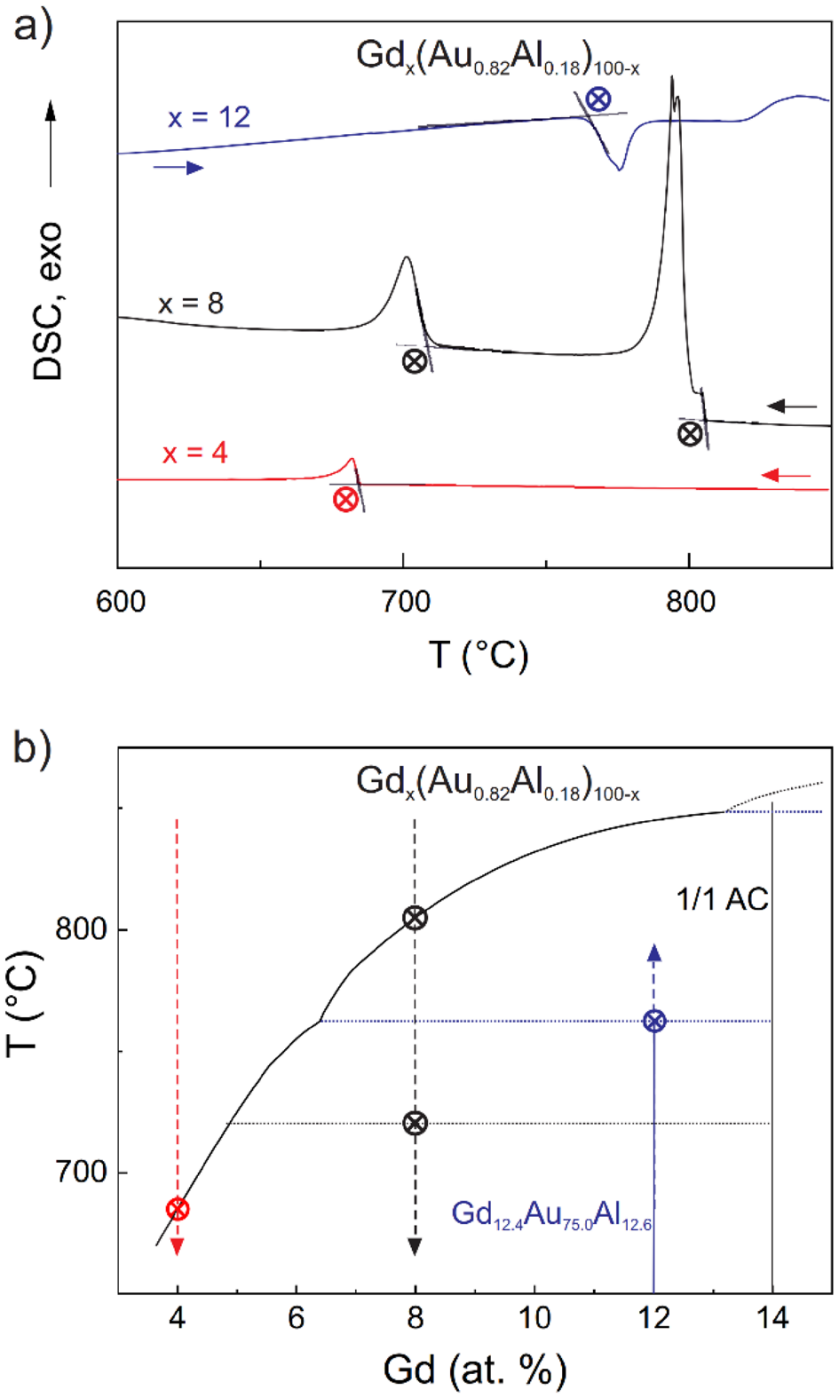
(**a**) DSC traces of samples Gd_x_(Au_0.82_Al_0.18_)_100−x_ with x = 4, 8 and 12. Actual temperatures of endothermic (x = 12) and exothermic (x = 4, 8) events are obtained by extrapolation of heating and cooling curves, respectively. x = 12 corresponds to an isolated grain of the title compound GdAu_6.75−x_Al_0.5+x_ (Gd_12.4_Au_75.0_Al_12.6_). (**b**) Sketch of the envisioned partial pseudobinary Gd–(Au_0.82_Al_0.18_) phase diagram. Note the discrepancy between peritectic formation and decomposition temperature (horizontal lines) for the title compound.

To explore the X-rich part of the Gd–(Au_0.82_Al_0.18_(X)) system a number of DSC experiments were undertaken. Figure 1a shows cooling curves of reaction mixtures Gd_x_(Au_0.82_Al_0.18_)_100−x_ with compositions x = 4, 8, and 12. There are two exothermic events for x = 8, at 800 and at 710 °C, which were interpreted as liquidus crossing (crystallization of AC phase) and peritectic formation (AC + liquid) of a new, more X-rich phase, respectively, thus rising expectations about the existence of a QC phase. Reducing the Gd concentration should then expose the liquidus for the new phase. Indeed, the DSC curve for x = 4 shows only one event, at 680 °C, corresponding to the crystallization of a new phase from the liquid. Accordingly, a synthesis experiment was performed by slowly cooling a melt with x = 4 to 600 °C and centrifuging off excess liquid. An analogous experiment targeting AC phase was employed with x = 8 and 750 °C as centrifugation temperature. Figure 1b shows a sketch of the envisioned pseudo-binary phase diagram.

**Figure 2:**
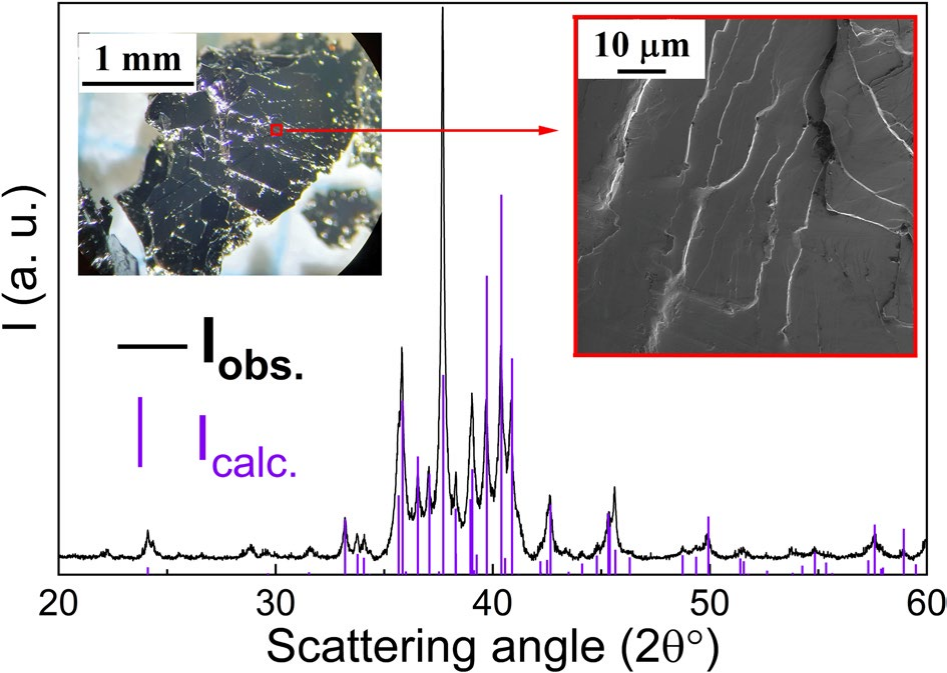
PXRD pattern of the GdAu_6.75−x_Al_0.5+x_ title compound (I_calc._ refers to the calculated intensity based on the structure obtained from SC-XRD). Diffraction background and peaks from Cu–K_α2_ radiation have been subtracted from I_obs._ for clarity. The inset shows an optical photograph (left) and SEM image (right) of GdAu_6.75−x_Al_0.5+x_ sample as it is obtained from synthesis; piles of sub-micron (< 1 µm) thick flakes, extended to large areas (mm^2^) were observed.

The phase obtained from the x = 4 solution growth experiment was afforded as larger than mm^2^-sized shiny flakes, which appeared to be built up from well crystalline plates with submicron sized thickness (inset in Fig. 2). Clearly, the pronounced lamellar appearance ruled out that the new phase represented a QC. The PXRD pattern (Fig. 2) shows a peculiar accumulation of the most intense diffraction peaks in a narrow 2Θ range 35°–42°. Relative peak intensities are clearly influenced by preferred orientation and could vary significantly depending on sample preparation condition (i.e. level of grinding). EDX analysis showed a homogenous composition, Gd_12.4(4)_Au_75.0(4)_Al_12.6(5)_, within single flakes and between flakes. The composition is remarkably similar to RE_12_X_88_ frequently found for Tsai-type QCs.

**Figure 3:**
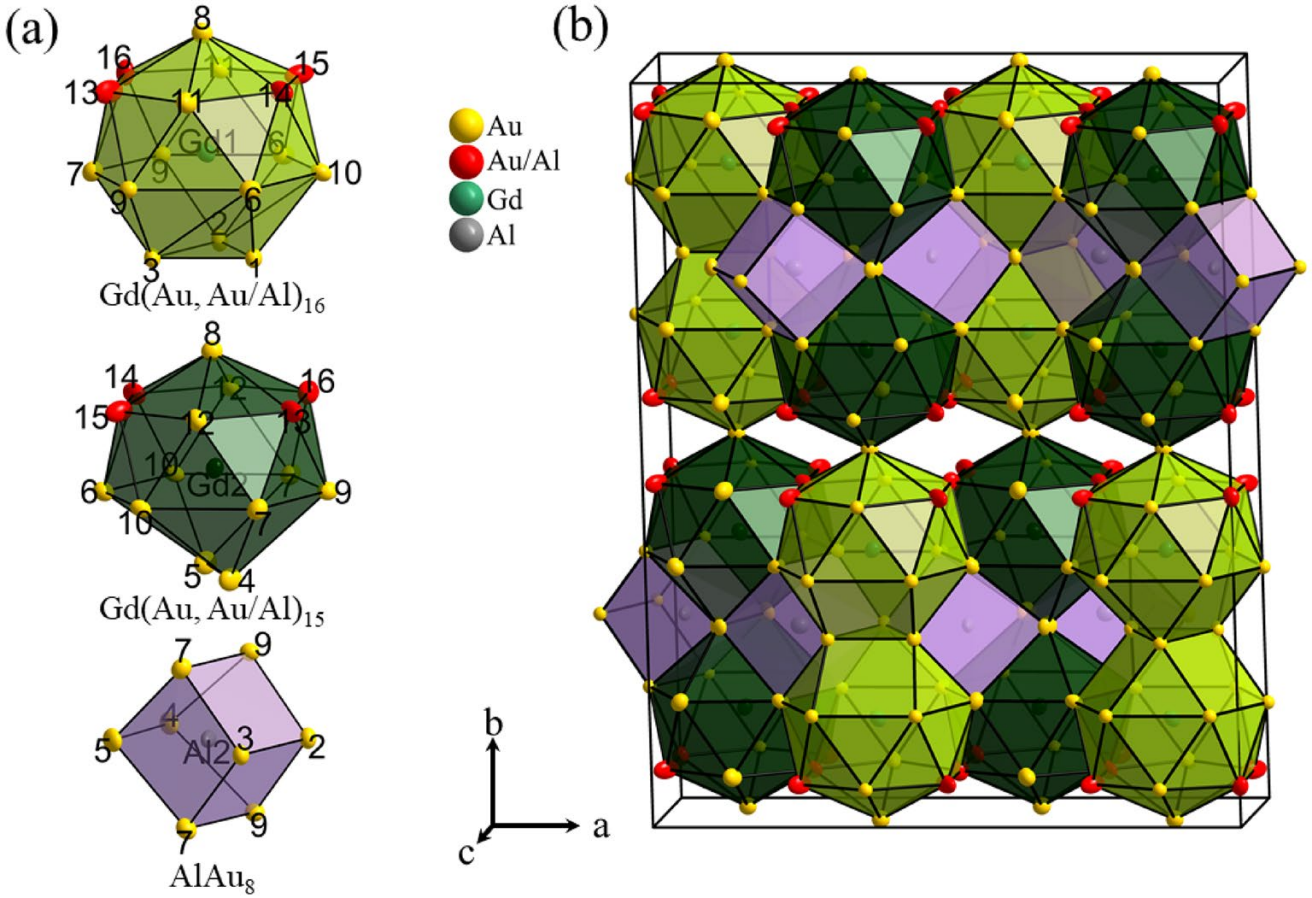
Crystal structure of GdAu_6.75−x_Al_0.5+x_. (**a**) Polyhedral units used to describe the structure. The polyhedra have been proportionally scaled with respect to each. (**b**) Arrangement of the polyhedra in the unit cell. All atomic positions are displayed by thermal ellipsoids at 90% probability.

**Figure 4:**
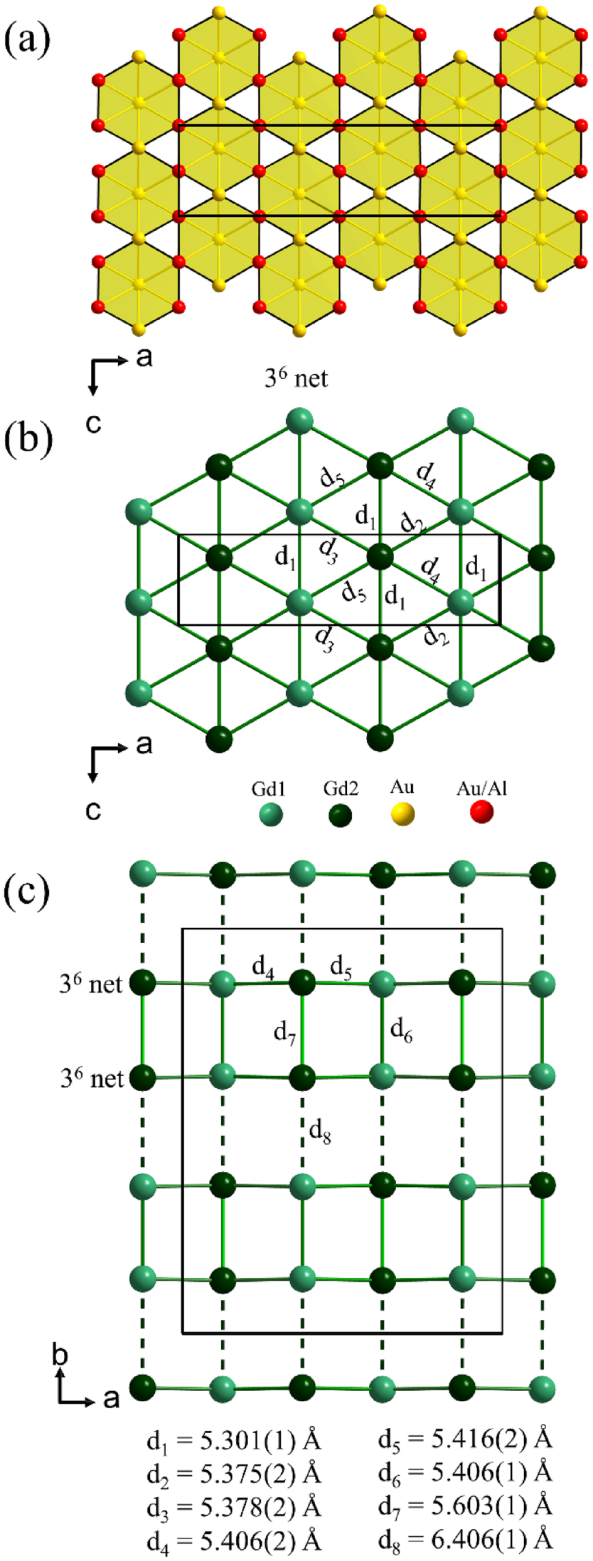
(**a**) Au8 coordination and Kagome nets formed by Au11, Au12 and (Au/Al)13–16 atoms. (**b**) Slightly corrugated 3^6^ net formed by Gd atoms in the *ac* plane. (**c**) Stacking of Gd nets along the *b* direction. Nearest interatomic Gd–Gd distances are listed. The unit cell is shown by a rectangle in each plot; the plots are proportionally scaled to each other.

The AC phase obtained from the x = 8 solution growth experiment was afforded as mm-sized rhombic-dodecahedral shaped crystals. EDX analysis yielded an expected composition Gd_15.0(4)_Au_70.9(5)_Al_14.1(3)_. The very similar Au/Al ratio of the AC phase and the new phase seems to validate the pseudo-binary approach. However, when performing a DSC heating–cooling cycle with the new phase (x = 12, Fig. 1a) peritectic decomposition occurs at around 750 °C, which is 50 °C higher than the peritectic formation temperature extracted from the x = 8 experiment. The PXRD pattern after the DSC heating–cooling cycle showed a mixture of the AC and the reformed new phase (Fig. S2 in the Supplemental Materials). This indicates that either the formation or decomposition of the new phase is strongly influenced by kinetics.

### Crystal structure of GdAu_6.75−x_Al_0.5+x_ (x ≈ 0.54(1))

Identifying suitable crystals of the new phase for SC-XRD proofed challenging. Single crystals were never perfect and frequently had small additional domains or were severely twinned. Patterns were indexed to a primitive orthorhombic lattice (a ≈ 18.8 Å, b ≈ 23.8 Å, c ≈ 5.3 Å) and the space group *Pnma* was assigned to the structure. Diffuse streaks along the h-direction signaled both positional and occupational disorder (Fig. S3). Among the positions obtained from the structure solution, Gd atoms were readily identified by Gd-X distances > 3 Å. Then the occupation factors were refined one at a time, indicating deviation from full occupancy for atoms Au13–Au16. For these positions Au/Al mixed occupancies were introduced with the constraint of equal position and ADP, as well as a fixed sum of occupancy (= 1.0), which finally yielded a composition of Gd_12.12_Au_75.3(1)_Al_12.5(1)_, in good agreement with the EDX analysis (Gd_12.4(4)_Au_75.0(4)_Al_12.6(5)_). The result of the structure refinement is summarized in Table 1; atomic position parameters are provided in Table 2, and selected interatomic distances are given in Table 3. Further crystallographic information can be found in the Supplemental Materials and in CCDC 2103496 on the Cambridge Crystallographic Data Centre (www.ccdc.cam.ac.uk/structures). The rather high value of residual density is both a consequence of disorder and a large number of low intensity reflections, which are prone to accumulate diffuse intensity. If only reflections with *I* ≥ 12σ(*I*) are considered, the crystallographic R-value reduces to R_1_ = 0.0184, wR_2_ = 0.0325 and GoF = 2.13 for 2164 independent reflections and the residual electron density down to + 2.73/− 2.11 eÅ^−3^, without any significant change to the structure.

**Table 1:** SC-XRD refinement and EDX results of GdAu_6.75−x_Al_0.5+x_.

Parameters	GdAu_6.75−x_Al_0.5+x_
Empirical formula	Gd_2_Au_12.43(2)_Al_2.07(2)_
Refined composition (at.%)	Gd_12.12_Au_75.3(1)_Al_12.5(1)_
EDX (at.%)	Gd_12.4(4)_Au_75.0(4)_Al_12.6(5)_
Formula weight	2819.2
Temperature/K	293
Crystal system	orthorhombic
Space group	Pnma
a/Å	18.7847 (4)
b/Å	23.8208 (5)
c/Å	5.30100 (10)
Volume/Å^3^	2372.02 (8)
Z	8
ρ_calc_ g/cm^3^	15.7885
μ/mm^−1^	89.408
F(000)	9096.0
Crystal size/mm^3^	0.138 × 0.097 × 0.045
Radiation	Ag K_α_ (λ = 0.56087)
2Θ range, data collection/°	2.7 to 49.86
Index ranges	− 28 ≤ h ≤ 28, − 35 ≤ k ≤ 35, − 7 ≤ l ≤ 7
Reflections collected	191,508
Ind. reflections [all data]	4287
Ind. reflections [I > = 3σ (I)]	3225
Merging R indices	R_int_ = 0.0663, R_sigma_ = 0.0158
Constraint/restraint/parameter	36/ 0/ 166
Goodness-of-fit GoF	2.700
Final R indexes [I > = 3σ (I)]	R_1_ = 0.0364, wR_2_ = 0.0552
Final R indexes [all data]	R_1_ = 0.0537, wR_2_ = 0.0585
Largest diff. peak/hole/e Å^−3^	6.93/− 6.85

**Table 2:** Atomic coordinates and equivalent atomic displacement parameters (U_eq._) of independent atomic positions for GdAu_6.75−x_Al_0.5+x_ obtained from SC-XRD refinement.

Atom	Wyck	S.O.F	x/a	y/b	z/c	U_eq._ [Å^2^]
Gd1	8d	1	0.12466 (6)	0.63653 (3)	0.25059 (12)	0.0110 (2)
Gd2	8d	1	0.12554 (6)	0.13240 (3)	0.25661 (12)	0.0110 (2)
Al1	4c	1	0.2330 (4)	0.25	0.4588 (13)	0.0135 (17)
Al2	4c	1	0.0182 (3)	0.25	0.0507 (14)	0.0135 (17)
Au1	4c	1	0.30269 (4)	0.25	0.91533 (14)	0.0107 (2)
Au2	4c	1	0.37525 (4)	0.25	0.42917 (14)	0.0112 (2)
Au3	4c	1	0.44895 (4)	0.25	0.91317 (15)	0.0112 (2)
Au4	4c	1	0.09639 (4)	0.25	0.49703 (15)	0.0145 (2)
Au5	4c	1	0.15522 (4)	0.25	0.01633 (15)	0.0147 (2)
Au6	8d	1	0.28774 (4)	0.15919 (3)	0.25412 (9)	0.0116 (2)
Au7	8d	1	0.04028 (4)	0.15902 (3)	0.7697 (1)	0.0127 (2)
Au8	8d	1	0.12486 (5)	0.50109 (2)	0.25097 (9)	0.0121 (2)
Au9	8d	1	0.46422 (4)	0.15897 (3)	0.25508 (9)	0.0111 (2)
Au10	8d	1	0.28982 (4)	0.65924 (3)	0.24258 (9)	0.0106 (2)
Au11	8d	1	0.37559 (5)	0.06020 (3)	0.2543 (1)	0.0158 (2)
Au12	8d	1	0.37484 (5)	0.55751 (3)	0.25893 (11)	0.0163 (2)
Au13	8d	0.912 (4)	0.00042 (4)	0.05792 (4)	0.50279 (14)	0.0188 (2)
Al13	8d	0.088 (4)	0.00042 (4)	0.05792 (4)	0.50279 (14)	0.0188 (2)
Au14	8d	0.772 (4)	0.25124 (4)	0.05744 (4)	0.00098 (15)	0.0165 (3)
Al14	8d	0.228 (4)	0.25124 (4)	0.05744 (4)	0.00098 (15)	0.0165 (3)
Au15	8d	0.715 (4)	0.24936 (4)	0.05787 (4)	0.50054 (15)	0.0160 (3)
Al15	8d	0.285 (4)	0.24936 (4)	0.05787 (4)	0.50054 (15)	0.0160 (3)
Au16	8d	0.533 (4)	0.00005 (5)	0.05586 (5)	0.00027 (18)	0.0133 (3)
Al16	8d	0.467 (4)	0.00005 (5)	0.05586 (5)	0.00027 (18)	0.0133 (3)

All atomic positions are normalized, Wyckhoff positions (Wyck.), site occupancy factors (S. O. F.) are listed and U_eq._ = 1/3(U_11_ + U_22_ + U_33_).

**Table 3:** Interatomic distances for GdAu_6.75−x_Al_0.5+x_ and GdAu_5.3_Al (1/1 AC) polyhedra obtained from SC-XRD refinement.

GdAu_6.75−x_Al_0.5+x_								GdAu_5.3_Al (1/1 AC)			
Atom pair			d/Å	Atom pair			d/Å	Atom pair			d/Å
Al1	Au4	1x	2.574 (7)	Gd1	Au7	1x	3.1461 (13)	Gd1	Au1	2x	3.1315 (11)
	Au6	2x	2.630 (4)		Au10	1x	3.1496 (13)		Au6	1x	3.1393 (12)
	Au10	2x	2.668 (4)		Au6	1x	3.1505 (10)		Au3|Al3	2x	3.1855 (16)
	Au2	1x	2.677 (7)		Au1	1x	3.1514 (9)		Au4	2x	3.1983 (13)
	Au1	1x	2.752 (7)		Au3	1x	3.1561 (9)		Au1	2x	3.2316 (13)
	Au5	1x	2.763 (7)		Au9	1x	3.1579 (10)		Au1	2x	3.2393 (9)
Al2	Au5	1x	2.580 (6)		Au6	1x	3.1818 (10)		Au3|Al3	1x	3.2400 (14)
	Au9	2x	2.606 (4)		Au2	1x	3.1952 (8)		Au2	2x	3.2733 (15)
	Au7	2x	2.662 (4)		Au9	1x	3.1977 (10)		Au2	1x	3.3267 (16)
	Au2	1x	2.688 (6)		Au11	1x	3.1980 (9)		Al5|Au5	1x	3.4695 (27)
	Au3	1x	2.782 (7)		Au8	1x	3.2262 (9)	Al8	Au4	2x	2.4922 (9)
	Au4	1x	2.785 (7)		Au11	1x	3.2304 (9)		Au1	6x	2.6340 (7)
Au8	Au12	1x	2.9585 (7)		Au13|Al13	1x	3.2767 (12)				
	Au11	1x	2.9857 (7)		Au14|Al14	1x	3.2780 (12)				
	Au14|Al14	1x	2.9957 (11)		Au15|Al15	1x	3.2967 (13)				
	Au16|Al16	1x	2.9970 (13)		Au16|Al16	1x	3.3089 (14)				
	Au13|Al13	1x	3.0123 (11)	Gd2	Au10	1x	3.0941 (10)				
	Au11	1x	3.0168 (7)		Au9	1x	3.0963 (13)				
	Au16|Al16	1x	3.0172 (13)		Au7	1x	3.1031 (10)				
	Au15|Al15	1x	3.0290 (12)		Au6	1x	3.1130 (13)				
	Au15|Al15	1x	3.0319 (12)		Au4	1x	3.1259 (8)				
	Au12	1x	3.0331 (7)		Au5	1x	3.1274 (8)				
	Au13|Al13	1x	3.0367 (11)		Au8	1x	3.1801 (9)				
	Au14|Al14	1x	3.0556 (11)		Au12	1x	3.1847 (9)				
	Gd2	1x	3.1801 (9)		Au15|Al15	1x	3.1990 (13)				
	Gd1	1x	3.2262 (9)		Au12	1x	3.2051 (9)				
					Au10	1x	3.2190 (9)				
					Au7	1x	3.2195 (10)				
					Au13|Al13	1x	3.2210 (12)				
					Au14|Al14	1x	3.2559 (12)				
					Au16|Al16	1x	3.2752 (14)				

The unit cell of the orthorhombic structure contains 132 atoms (Pearson symbol oP132) which are distributed on 20 positions, 2 corresponding to Gd, 12 to Au, 2 to Al, and 4 to mixed occupied Au/Al. The crystallographic composition is GdAu_6.75−x_Al_0.5+x_ (x ≈ 0.54(1)) (GdX_7.25_), and hereon we refer to the new phase as GdAu_6.75−x_Al_0.5+x_. Its structure can conveniently be described using three polyhedral units as shown in Fig. 3a. The two Gd atoms are coordinated by 16 (Gd1) and 15 (Gd2) atoms, respectively. The Gd-(Au, Al) distances are in a quite narrow range, between 3.09 and 3.31 Å (Table 3). The Gd2(Au, Al)_15_ and Gd1(Au, Al)_16_ polyhedra are based on hexagonal antiprisms, which are capped on one side by either a triangle (16-atom polyhedra) or a pair of atoms (15-atom polyhedra) and on the opposite side by a single atom (corresponding to the Au8 position for both). Like polyhedra are fused to pairs by shared triangles and atom-pairs, respectively, and polyhedron pairs are condensed to layers in the *ac* plane in a close-packed (3^6^) fashion (Fig. 3b). Al atoms are located at the center of these layers, at y = ¼ and ¾, and attain a quasi-cubic coordination by 8 Au atoms (Fig. 3a). Al–Au distances are in a range 2.57–2.79 Å (Table 3). Finally, layers of polyhedron pairs are then connected via the single capping atom (Au8) along the *b* direction, at *y* ≈ 0 and ½, which gives the structure a pronounced two dimensional character.

The mixed occupied positions (Au/Al)13–16 are concentrated in two Kagome nets which are situated above and below the layer connecting Au atoms which provides them with a hexagonal prismatic coordination environment (Fig. 4a). For the later discussion of the magnetic properties of GdAu_6.75−x_Al_0.5+x_ we also highlight the Gd partial structure. The Gd atoms are arranged as slightly corrugated 3^6^ nets, which are stacked on top of each other (Fig. 4b). Gd–Gd distances within nets are between 5.30 and 5.41 Å. The distance between two nets within a polyhedral layer is ~ 5.5 Å and in between 6.4 Å (Fig. 4c). Thus the stacking of Gd 3^6^ nets follows a “short”- “long” pattern.

### Magnetic properties of GdAu_6.75−x_Al_0.5+x_

**Figure 5:**
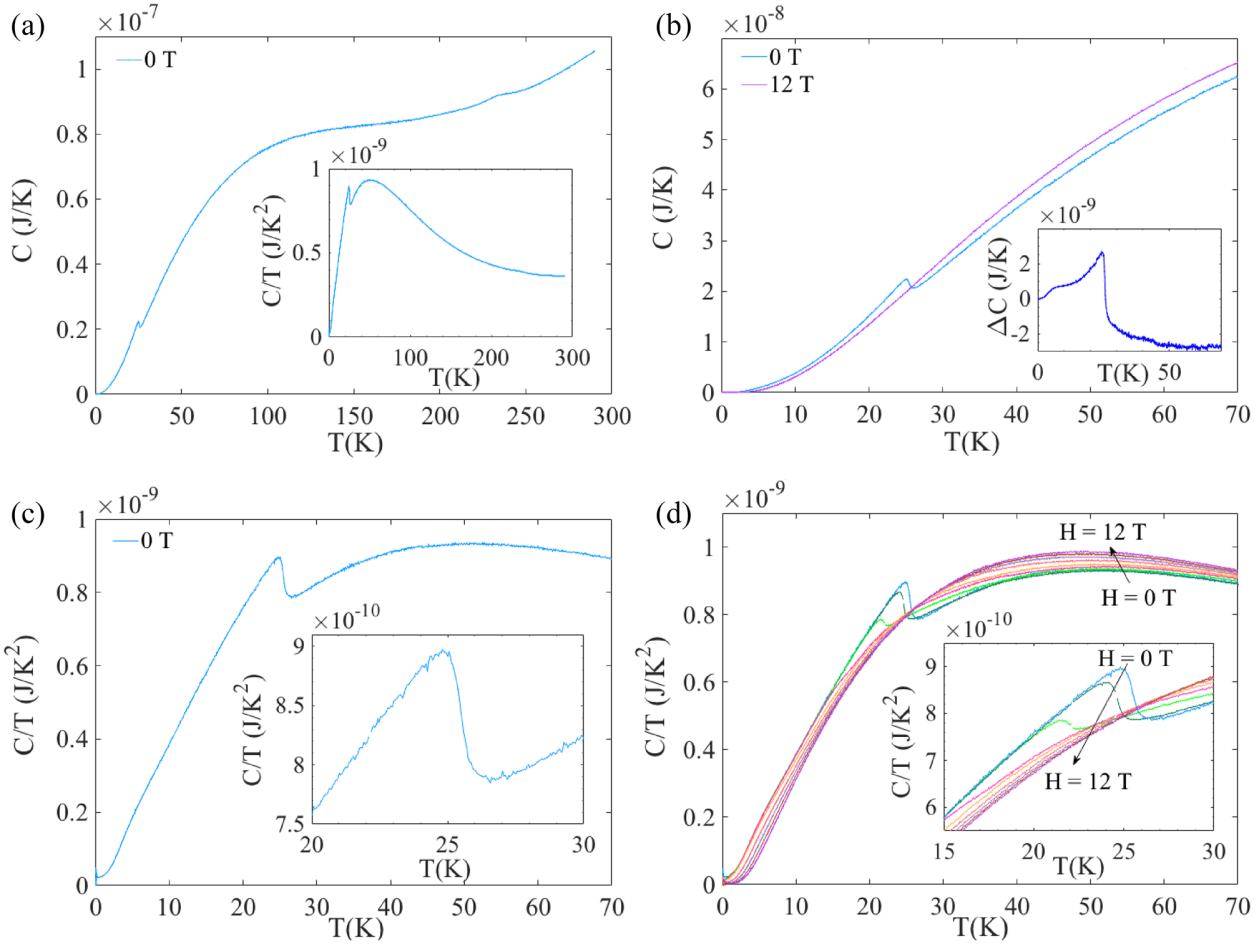
(**a**) Temperature dependence of Specific heat C(T) of GdAu_6.75−x_Al_0.5+x_ recorded under zero magnetic field. Inset shows the C/T versus T plot of the main panel. (**b**) C(T) curves recorded under two extreme magnetic fields i.e. H = 0 T and H = 12 T; their difference ΔC is plotted in the inset. C/T versus T curves for (**c**) H = 0 T, (**d**) H = 0, 1, 2, 3, 4, 6, 8, 10 and 12 T. Insets of bottom panels show the zoomed view of corresponding main figure.

Figure 5 shows the heat capacity C data recorded as a function of temperature and magnetic fields. The measured C vs T curves recorded in zero and maximum magnetic field (12 T) are plotted in panels (a) and (b). A significant difference is observed in the curves recorded with and without applied magnetic field. A peak, more clearly observed in the C/T(T) curves plotted in panel (c), can be seen at about 25 K, suggesting a magnetic transition. As seen in panels (d), the heat capacity is significantly affected by magnetic fields, with the transition being shifted to lower temperatures, and smeared out in relatively moderate magnetic fields. The high temperature data (above 25 K) in panels (b) and (d), suggests that a magnetic field larger than 12 T is required to determine the non-magnetic baseline of the heat capacity and analyze further the temperature and magnetic field dependence of the heat capacity data of GdAu_6.75−x_Al_0.5+x_.

**Figure 6:**
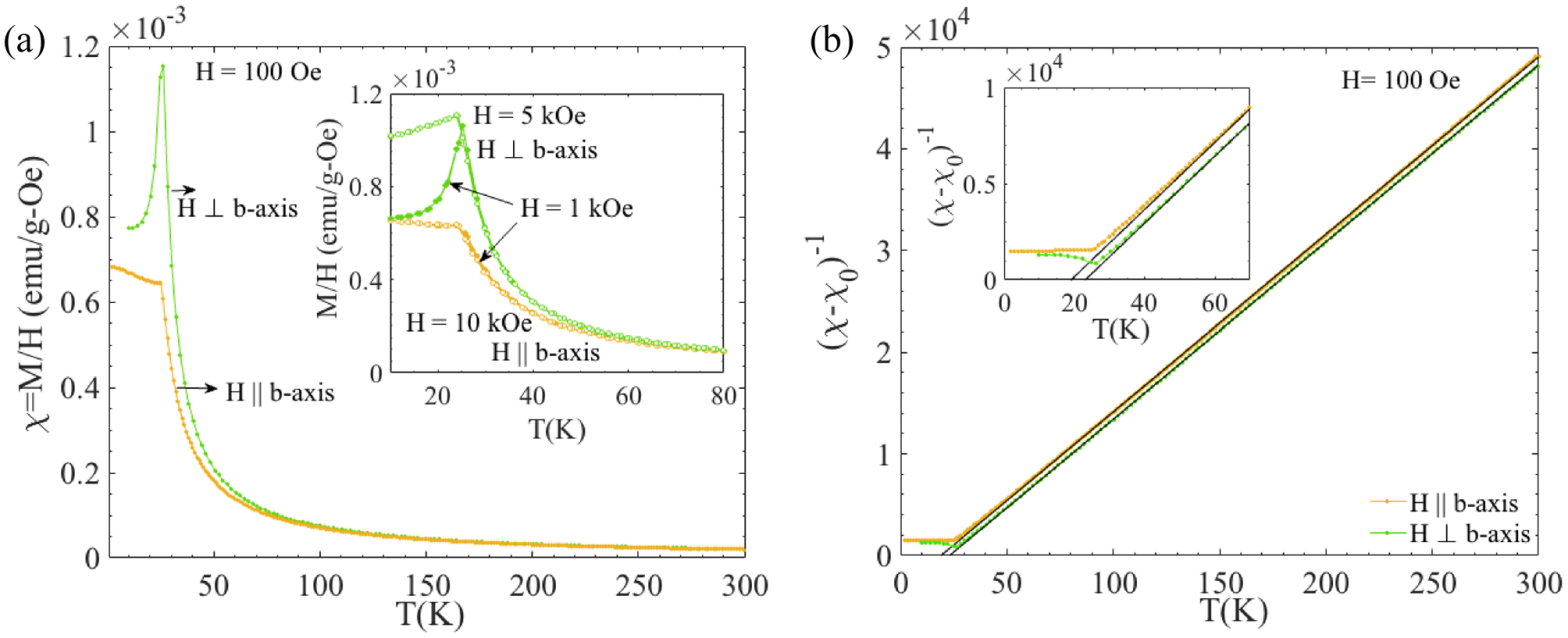
(**a**) Temperature dependence of magnetic susceptibility χ(T) of GdAu_6.75−x_Al_0.5+x_ measured after ZFC with H = 100 Oe along H⊥*b*-axis (green color) and H∥*b*-axis (yellow color). The inset shows the χ(T) curve recorded after ZFC and FC at relatively higher magnetic fields for both orientations. (**b**) Curie–Weiss plots using temperature dependence of the inverse magnetic susceptibility for both orientations, where χ_0_ is the background correction term. The black solid lines are the best fits of (χ − χ_0_)^−1^(T) to the Curie–Weiss equation. Inset shows the zoomed view of the main panel.

Figure 6a shows the temperature dependence of magnetization M(T) measured after ZFC in H = 100 Oe for GdAu_6.75−x_Al_0.5+x_ along H⊥*b*- and H∥*b*-axis. The overall behavior of the M(T) curves is reminiscent of long-range ferromagnetic behavior. The susceptibility (M(T)/H) at high temperatures follows a Curie–Weiss (CW) law for both directions as shown in Fig. 6b and yields the Curie–Weiss temperatures θ_CW⊥_ ~  + 23 and θ_CW∥_ ~  + 19 K, respectively for H⊥*b*- and H∥*b*-axis with an effective moment µ_eff_ ~ 8.1 µ_B_ for both the orientations. The observed value of the effective moment is close to the expected µ_eff_ of Gd^3+^ ($g\sqrt{J\left(J+1\right)}=$ 7.94 µ_B_).

A peak is observed for H⊥*b*-axis near 25 K, i.e. in the vicinity of the temperature of the peak observed in the heat capacity data. For H∥*b* the susceptibility data below 26 K is slowly increasing with decreasing temperature. As seen in the inset of the Fig. 6a, the ZFC/FC curves are reversible in the whole temperature range. The data collected with H = 1 kOe is qualitatively similar to that collected in a smaller magnetic field in both directions (see main frame). With H = 5 kOe, the susceptibility does not decrease as rapidly with decreasing temperature when H⊥*b*, suggesting a non-linear increase of the magnetization in that case. For H∥*b* the susceptibility is not significantly affected, even for H = 10 kOe.

**Figure 7:**
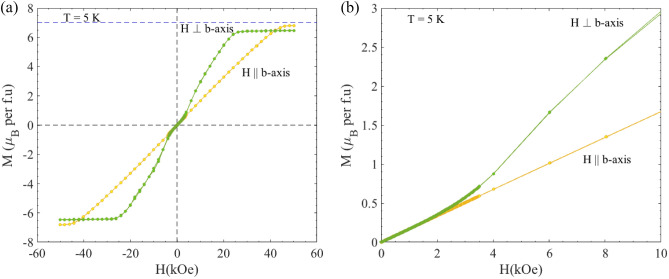
(**a**) M(H) curves for GdAu_6.75−x_Al_0.5+x_ recorded at a constant temperature T = 5 K for H⊥*b*-axis and H∥*b*-axis. (**b**) Zoomed view of (**a**) to observe the change in linearity for H⊥*b*-axis at low fields. To clearly notice these changes, a small step size (ΔH = 20 Oe) was used within the field interval -3500 Oe ≤ H ≤ 3500 Oe during the measurement.

Figure 7 shows the magnetic field dependence of the magnetization M(H) recorded at T = 5 K, for both orientations. The magnetization increases with the magnetic field until it saturates at 26 kOe and 44 kOe for H⊥*b*-axis and H∥*b*-axis, respectively. The observed value of the saturation moment M_sat_ ~ 7µ_B_/Gd is similar to that of Gd^3+^ (*gJ* = 7.00 µ_B_). No hysteresis is observed as the field is swept in reverse direction. While the low-field M(H) curve is linear for H∥*b*, a close inspection of the H⊥*b*-axis M(H) curves reveals a change in the slope in the M(H) curves near 3.4 kOe. This is consistent with the behavior of the temperature-dependent susceptibility curves reported below and above 3.4 kOe (1 and 5 kOe) for H⊥b (see inset of Fig. 6a).

The positive value of the Curie–Weiss temperature(s) reflects the dominance of the FM interaction. However the lack of irreversibility in the ZFC/FC M(T) curves and the non-hysteretic behavior of the low-field M(H) curves suggest an antiferromagnetic response of the system, whose magnetic moments may be reoriented by a sufficiently large applied magnetic field. In spite of a different origin and nature for the magnetic interaction for 3d systems with covalent magnetism^17^ and 4f. intermetallic systems with RKKY interaction^18^, several type-A antiferromagnets such as (3d) NaNiO_2_^19–21^, FeTiO_3_^22^, and (4f.) EuRh_2_Si_2_^23^ show similar magnetic characteristics as GdAu_6.75−x_Al_0.5+x_. For example, NaNiO_2_ exhibits a sharp peak below 20 K marking an antiferromagnetic transition with a positive Curie–Weiss constant θ_CW_ =  + 36 K^19–21^ (i.e. a positive value). In that system, the low-temperature M(H) curve saturates around 120 kOe with a spin flop transition near 18 kOe^19–21^. NaNiO_2_ consists of a triangular layered crystal structure where the ferromagnetic planes are coupled antiferromagnetically to the alternate ferromagnetic planes^19^. This suggests that the magnetic state of GdAu_6.75−x_Al_0.5+x_ is composed of ferromagnetic planes (positive Curie–Weiss temperatures), antiferromagnetically coupled to each other (see Supplemental Materials for a sketch of the spin configuration). The linear low-field M(H) up to saturation in the H∥b case suggests that the magnetic moments lie perpendicularly to that direction. The slope change observed in the low-field M(H) for H⊥b may hence reflect a spin-flop of the magnetic moments; the initial orientation of the magnetic moments and magnetic field in the *ab*-plane being unknown. Such magnetic structure is consistent with the crystal structure determined above, which include 3^6^ *ac*-planes of Gd cations (see Fig. 4). We speculate that those planes are ferromagnetic, and antiferromagnetically coupled to each other. Such a magnetic structure is consistent with the magnetic field dependence of the heat capacity data presented in Fig. 5d. Interestingly the present system provides a new example of intermetallic compound with complex crystal structure and magnetic anisotropy^24^ which may be used as a reference or model system when investigating magnetic properties or/and structure–property relationships, e.g. in approximant crystals as illustrated below.

### Comparison to the structural and magnetic properties of approximant crystals

**Figure 8:**
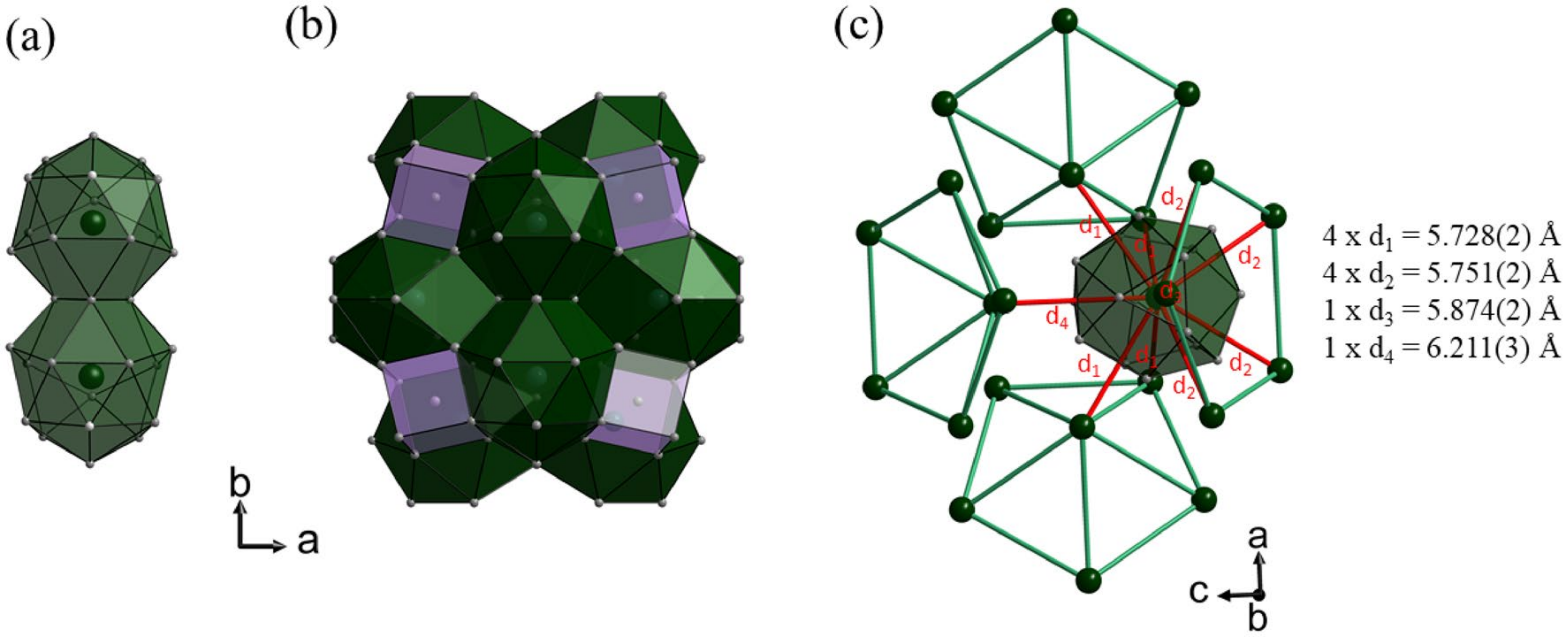
Crystal structure of the 1/1 AC. (**a**) Pair of Gd(Au/Al)_16_ polyhedra condensed via a common triangle, (**b**) arrangement of Gd(Au/Al)_16_ polyhedra and AlAu_8_ cubes in the unit cell and (**c**) Gd partial structure. Nearest Gd–Gd interatomic distances are drawn as red lines and their numbers are listed. The green lines highlight the connectivity of Gd atoms into icosahedral shells in Tsai clusters.

Although at first sight not apparent, one may discern a relation to the 1/1 approximant crystal structure (Fig. 8) when focusing on the Gd(Au, Al)_16_ polyhedra as building units instead of the more commonly used Tsai-type clusters. We have analyzed a crystal obtained from the x = 8 solution growth experiment by SC-XRD. The refinement result corresponded virtually to the one reported by Ishikawa et al. for Gd_14_Au_73_Al_13_ obtained from arc-melting synthesis^10^. The refined composition of our 1/1 AC crystal was Gd_13.6_Au_72.8(3)_Al_13.6(3)_ (GdAu_5.3_Al).

In the cubic 1/1 AC structure (space group *Im*-3) there is only one type of Gd atom at the (0.00, 0.185, 0.303) crystallographic position (on a 24 g site) which is coordinated by 16 (Au/Al) atoms at distances between 3.13 and 3.47 Å (cf. Table 3). This 16 atom polyhedron has been earlier described as mono-capped, double, pentagonal antiprism^25^ and is thus different to the Gd1(Au, Al)_16_ polyhedron in GdAu_6.75−x_Al_0.5+x_. Nevertheless, the connection to neighboring polyhedra via shared triangle faces resembles the GdAu_6.75−x_Al_0.5+x_ structure, and a pair of polyhedra is shown in Fig. 8a. In addition, cubic interstitials (at the position 8c) are filled by Al atoms at the (0.25, 0.25, 0.25) crystallographic position and coordinated by 8 Au atoms at distances between 2.49 and 2.63 Å (cf. Table 3) forming slightly distorted cubes with an edge length of ~ 3 Å (Fig. 8b). One may speculate that the formation of both GdAu_6.75−x_Al_0.5+x_ and 1/1 AC phase is initiated by Gd(Au, Al)_n_ polyhedra. These polyhedra may then represent seeds for the growth of the concentric shells of Tsai clusters (“curling”) or arranged into 2D layers. The curling vs planar arrangement of intermetallic structure units has been discussed earlier in terms of chemical pressure^26^.

Figure 8c displays the partial Gd structure of the 1/1 AC. In contrast with the 2D 6 + 1 arrangement in GdAu_6.75−x_Al_0.5+x_ each Gd atom is surrounded by 9 + 1 neighboring Gd atoms. The distances to 9 neighbors are in a narrow range between 5.72 and 5.87 Å (and thus somewhat larger than the Gd–Gd distances within the layer of GdAu_6.75−x_Al_0.5+x_). The 10th distance is at 6.21 Å and thus somewhat shorter the Gd–Gd distance between layers in GdAu_6.75−x_Al_0.5+x_ (cf. Fig. 4b,c).

The here reported temperature and field dependences of the magnetization of GdAu_6.75−x_Al_0.5+x_ indicating type-A antiferromagnetic structure, shows similarities with those of certain approximant crystals. Ishikawa et al. reported antiferromagnetic transitions for the Gd_14_Au_73_Al_13_ and Tb_14_Au_72_Al_14_ quasicrystal approximants^10^; they also observed metamagnetic features in the M(H) curves of the materials, which may be related to their complex magnetic structures^11,27^. The Curie–Weiss analysis yielded positive values of θ_CW_ viz. + 5.9 K and + 4.2 K for Gd_14_Au_73_Al_13_ and Tb_14_Au_72_Al_14_, respectively^10^. Similar magnetization behavior was reported for Eu_14_Au_66_Ga_20_ and Eu_14.5_Au_65_Ga_20.5_ (Eu–Au–Ga) with antiferromagnetic transition temperatures of 7 K and 8.5 K and negative values of θ_CW_ = − 4.45 K and − 1.70 K, respectively^28^. The magnetic ordering temperatures in all these approximants were confirmed by zero-field temperature dependent specific-heat C(T) data^10,28^, where quite sharp lambda-like anomalies appear at the magnetic ordering temperatures indicated by the M(T) curves. Both RE-Au–Al and Eu–Au–Ga systems were found to show metamagnetic-like behavior^10,28^, albeit such effects are much weaker in the case of Eu–Au–Ga^28^, whose M(H) curves show similarities with the present GdAu_6.75−x_Al_0.5+x_ system.

## Conclusion

The Au rich end of the ternary Gd–Au–Al system was investigated for which a simplified pseudo-binary approach with two components Gd and Au_82_Al_18_ was followed. The self-flux syntheses method was employed and resulted to a new intermetallic compound GdAu_6.75−x_Al_0.5+x_ (*Pnma*, a = 18.7847(4) Å, b = 23.8208(5) Å, c = 5.3010(1) Å) ascertained by SC-XRD refinement. GdAu_6.75−x_Al_0.5+x_ crystallizes in a complex new structure with two-dimensional character in which the Gd atoms are arranged in 3^6^ nets (d_Gd–Gd_ = 5.30–5.41 Å) which are stacked on top of each other along the *b* direction. The structure of GdAu_6.75−x_Al_0.5+x_ bears some relation with those of approximant crystals. Akin to some of those systems, the magnetization curves of GdAu_6.75−x_Al_0.5+x_ were found to display sharp peaks associated with magnetic ordering, and metamagnetic-like transitions. The material becomes antiferromagnetic below 25 K; the magnetometry results suggest that the antiferromagnetic state is composed of ferromagnetic *ac* planes, coupled antiferromagnetically along the *b* direction. This suggests that the 3^6^ *ac* planes are ferromagnetic, and antiferromagnetically coupled along the *b* stacking direction below the magnetic ordering temperature at 25 K.

## Methods

### Synthesis and structucal properties

Starting materials were granules of the elements Au (Chempur, 99.99%), Al (SigmaAldrich 99.999%) and Gd (Chempur, 99.99%). Prior the synthesis reactions, Au and Al were arc-melted in a ratio 82:18 (at. %) to produce an (inhomogeneous) “alloy” or “pseudoelement” X = Au_0.82_Al_0.18_. Actual reaction mixtures of constituted compositions were Gd_x_(Au_0.82_Al_0.18_)_100−x_ with x in the range 4–12. Reaction mixtures were investigated with Differential Scanning Calorimetry (DSC) prior to the solution-growth synthesis to extract liquidus temperatures. Synthesis targeting the AC phase Gd_14_X_86_ and potentially QC phase Gd_12_X_88_ were performed with x = 8 and 4, respectively. For synthesis reactions alumina (Al_2_O_3_) crucibles from LSP Industrial Ceramics (USA) were employed, in the form of ‘Canfield Crucible Sets (CCS)’. The CCS consists of two flat bottom cylindrical crucibles and an alumina frit-disc with holes of ~ 0.7 mm to 1 mm in diameter designed to separate solid grain from liquid melt during centrifugation^29^. A total mass of 3 g was weighed inside a glove box (Ar-atmosphere, < 0.1 ppm O_2_), loaded into the CCS which was then encapsulated inside a stainless-steel ampule. Ampules were heated in a commercial multi-step programmable muffle furnace to 1000 °C and dwelled for 10 h for achieving homogenous melts. Subsequently, the temperature was lowered to 600 °C and 750 °C for x = 4 and 8, respectively, using a cooling rate of 1 °C/h, and reactions terminated by isothermally centrifuging off excess melt at the target temperatures (see Fig. S1 in the Supplemental Materials for the synthesis temperature profile).

Powder X-ray diffraction (PXRD) data were collected on a Bruker D8-POWDER diffractometer with θ–2θ diffraction geometry and a Cu–K_α_ radiation (K_α1_ = 1.540598 Å and K_α2_ = 1.544390 Å) at room temperature. The powdered sample was applied to a zero-diffraction plate and diffraction pattern was measured in a 2θ range of 5°–90°. PXRD data were analyzed with the HighScore Plus 3.0 software from PANalytical^30^.

Single crystal X-ray diffraction (SC-XRD) data was collected on two diffractometers from Bruker, SMART-APEX and D8, both using an Incoatec microfocus X-ray source with λ = 0.56087 Å and 0.71073 Å, respectively and an APEXII CCD area detector. A prism-shaped and a fragmented crystal with a metallic luster was mounted on a MiTeGen Micromount and a Cactus needle using small amounts of perfluorinated polyakylether and Epoxy glue, respectively. Due to the occupational disorder in combination with the high absorption coefficient, the dataset was measured to a high degree of redundancy. Data reduction and numerical absorption correction was performed with SADABS 2014/2^31^. The structures of the title compound and the AC phase were solved by charge-flipping (Superflip^32^) and refined in JANA2006^33^ in the space groups *Pnma* (#62) and Im-3 (#204), respectively.

Microstructures were visualized by optical and scanning electron microscopy (SEM). A Zeiss-Merlin SEM instrument equipped with X-Max 80 mm^2^ Silicon Drift energy dispersive X-ray (EDX) detector with high sensitivity and at high count rates was employed for compositional analysis. Prior to the EDX experiment samples were mechanically polished using Silicon carbide coated papers. EDX data was collected with an acceleration voltage of 20 kV over larger areas (~ 1 × 1 mm) on at least 20 points.

DSC measurements were performed with a NETZSCH STA 449 F1 Jupiter instrument. Sample specimen with a total mass of ~ 100 mg were placed in a polycrystalline sapphire crucible (outer diameter = 5 mm, thickness = 0.5 mm) and a heating/cooling cycle to 1150 °C was performed at a rate of 10 °C/minute under an Ar flow of ~ 40 mL/min. An empty crucible served as reference.

### Physical properties

The magnetic properties of a thin flake shape of GdAu_6.75−x_Al_0.5+x_ with dimensions ≤ 50 µm × 2–3 mm^2^ oriented along *ac*-plane i.e., perpendicular to the *b*-axis, were recorded using a superconducting quantum interference design (SQUID) from Quantum Design Inc. The magnetic field H was applied along two different orientations of the sample (1) perpendicular to the b-axis (H⊥b) and (2) parallel to the b-axis (H∥b). The temperature dependence of the magnetization M(T) was recorded in zero field cooled (ZFC) and field cooled (FC) conditions in different magnetic fields. The field dependence of the magnetization M(H) was recorded at T = 5 K. The heat capacity C(T,H) data were collected on a flake with dimensions ≤ 50 μm × (50 × 50) μm^2^ as a function of temperature and magnetic fields down to ∼ 100 mK using a differential membrane-based nanocalorimeter^34^ and a Bluefors dilution refrigerator equipped with a superconducting magnet. Background corrections from the calorimeter membrane and Apiezon grease used to attach the sample were performed on the measured data (See Supplemental Materials).

## Supplementary Information

Supplementary Information.

## Data Availability

The datasets used and/or analysed during the current study available from the corresponding author on reasonable request.

## References

[1] Goldman A (2014). Magnetism in icosahedral quasicrystals: Current status and open questions. Sci. Technol. Adv. Mater..

[2] Tamura R, Ishikawa A, Suzuki S, Kotajima A, Tanaka Y, Seki T, Shibata N, Yamada T, Fujii T, Wang C-W, Avdeev M, Nawa K, Okuyama D, Sato TJ (2021). Experimental observation of long-range magnetic order in icosahedral quasicrystals. J. Am. Chem. Soc..

[3] Lifshitz R (1998). Symmetry of magnetically ordered quasicrystals. Phys. Rev. Lett..

[4] Suzuki S, Ishikawa A, Yamada T, Sugimoto T, Sakurai T, Tamura R (2021). Magnetism of Tsai-type quasicrystal approximants. Mater. Transac..

[5] Tsai A-P, Guo J, Abe E, Takakura H, Sato T (2000). A stable binary quasicrystal. Nature.

[6] Takakura H, Pay Gómez C, Yamamoto A, De Boissieu M, Tsai A-P (2007). Atomic structure of the binary icosahedral Yb–Cd quasicrystal. Nat. Mater..

[7] Goldman A, Kelton R (1993). Quasicrystals and crystalline approximants. Rev. Mod. Phys..

[8] Gebresenbut G, Tamura R, Eklöf D, Pay Gómez C (2013). Syntheses optimization, structural and thermoelectric properties of 1/1 Tsai-type quasicrystal approximants in RE–Au–SM systems (RE = Yb, Gd and SM = Si, Ge). J. Phys. Condens. Matter..

[9] Ishikawa A, Hiroto T, Tokiwa K, Fujii T, Tamura R (2016). Composition-driven spin glass to ferromagnetic transition in the quasicrystal approximant Au–Al–Gd. Phys. Rev. B.

[10] Ishikawa A, Fujii T, Takeuchi T, Yamada T, Matsushita Y, Tamura R (2018). Antiferromagnetic order is possible in ternary quasicrystal approximants. Phys. Rev. B.

[11] Sato T, Ishikawa A, Sakurai A, Hattori M, Avdeev M, Tamura R (2019). Whirling spin order in the quasicrystal approximant Au_72_Al_14_Tb_14_. Phys. Rev. B.

[12] Goldman A, Kong T, Kreyssig A, Jesche A, Ramazanoglu M, Dennis K, Bud'ko S, Canfield P (2013). A family of binary magnetic icosahedral quasicrystals based on rare earths and cadmium. Nat. Mater..

[13] Reichmann TL, Ipser H (2014). Reinvestigation of the Cd–Gd phase diagram. J. Alloys. Compds..

[14] Canfield P, Caudle M, Ho C-S, Kreyssig A, Nandi S, Kim M, Lin X, Kracher A, Dennis K, McCallum R (2010). Solution growth of a binary icosahedral quasicrystal of Sc_12_Zn_88_. Phys. Rev. B.

[15] Gebresenbut G, Eklöf D, Gordeeva A, Shiino T, Häussermann U (2021). Peritectic formation and phase stability of the icosahedral quasicystal i-GdCd and its ternary variants with Zn, Mg, and Y. Cryst. Growth Des..

[16] Murray JL, Okamoto H, Massalski TB, Okamoto H, Schlesinger ME, Mueller EM (1987). Al–Au phase diagram. Binary Phase Diagrams, Alloy Phase Diagram, Vol 3, ASM Handbook.

[17] Goodenough JB, Cotton FA (1963). Magnetism and the chemical bond. Interscience Monographs on Chemistry.

[18] Sugimoto T, Tohyama T, Hiroto T, Tamura R (2016). Phenomenological magnetic model in Tsai-type approximants. J. Phys. Soc. Jpn.

[19] Darie C, Bordet P, De Brion S, Holzapfel M, Isnard O, Lecchi A, Lorenzo J, Suard E (2005). Magnetic structure of the spin-1/2 layer compound NaNiO_2_. Eur. Phys. J. B.

[20] Borgers P, Enz U (1966). Metamagnetism of NaNiO_2_. Solid State Commun..

[21] Holzapfel M, De Brion S, Darie C, Bordet P, Chappel E, Chouteau G, Strobel P, Sulpice A, Núñez-Regueiro M (2004). Decoupling of orbital and spin degrees of freedom in Li_1__−__x_Na_x_NiO_2_. Phys. Rev. B.

[22] Stickler JJ, Kern S, Wold A, Heller G (1967). Magnetic resonance and susceptibility of several ilmenite powders. Phys. Rev..

[23] Seiro S, Geibel C (2014). Complex and strongly anisotropic magnetism in the pure spin system EuRh_2_Si_2_. J. Phys. Condens. Matter.

[24] Rotter M, Loewenhaupt M, Doerr M, Lindbaum A, Sassik H, Ziebeck K, Beuneu B (2003). Dipole interaction and magnetic anisotropy in gadolinium compounds. Phys. Rev. B.

[25] Pay Gómez C, Lidin S (2003). Comparative structural study of the disordered *M*Cd_6_ quasicrystal approximants. Phys. Rev. B.

[26] Berns VM, Fredrickson DC (2013). Problem solving with pentagons: Tsai-type quasicrystal as a structural response to chemical pressure. Inorg. Chem..

[27] Miyazaki H, Sugimoto T, Morita K, Tohyama T (2020). Magnetic orders induced by RKKY interaction in Tsai-type quasicrystalline approximant Au–Al–Gd. Phys. Rev. Mater..

[28] Yoshida S, Suzuki S, Yamada T, Fujii T, Ishikawa A, Tamura R (2019). Antiferromagnetic order survives in the higher-order quasicrystal approximant. Phys. Rev. B.

[29] Canfield P, Kong T, Kaluarachchi U, Jo NH (2016). Use of frit-disc crucibles for routine and exploratory solution growth of single crystalline samples. Philos. Mag..

[30] Degen T, Sadki M, Bron E, König U, Nénert G (2014). The HighScore suite. Powder Diffr..

[31] Bruker (2014). SADABS; Version 2014/2.

[32] Palatinus L, Chapuis G (2007). SUPERFLIP–a computer program for the solution of crystal structures by charge flipping in arbitrary dimensions. J. Appl. Cryst..

[33] Petříček V, Dušek M, Palatinus L (2014). Crystallographic computing system JANA2006: General features. Zeit. Krist. Cryst. Mater..

[34] Tagliati S, Krasnov VM, Rydh A (2012). Differential membrane-based nanocalorimeter for high-resolution measurements of low-temperature specific heat. Rev. Sci. Instrum..

