# Supplementary material for: 2D crystal structure and anisotropic magnetism of GdAu6.75−xAl0.5+x (x ≈ 0.54)
Source: Sci Rep. 2022 Jul 30;12:13141. doi: 10.1038/s41598-022-17068-4 (PMC9338926; doi:10.1038/s41598-022-17068-4)
Supplement: Supplementary file 1 — Supplementary Information. [file 41598_2022_17068_MOESM1_ESM.pdf]

Supplemental Materials

for

2D crystal structure and anisotropic magnetism of $GdAu_{6.75-x}Al_{0.5+x}$ ($x \approx 0.54$)

D. C. Joshi[†1*], G. H. Gebresenbut[†2], A. Fischer[†3], A. Rydh[4], U. Häussermann[5], P. Nordblad[1], R. Mathieu[1*]

[1]*Department of Materials Science and Engineering, Uppsala University, Box 35, SE-751 03, Uppsala, Sweden*

[2]*Department of Chemistry-Ångström laboratory, Uppsala University, 751 21 Uppsala, Sweden*

[3]*Institute of Physics, Augsburg University, 86159 Augsburg, Germany*

[4]*Department of Physics, Stockholm University, SE-106 91 Stockholm, Sweden*

[5]*Department of Materials and Environmental Chemistry, Stockholm university, 106 91 Stockholm, Sweden*

[†]These authors contribute equally to this work

*Corresponding authors: djoshi835@gmail.com; roland.mathieu@angstrom.uu.se

Table of contents:

S1. Synthesis details for $GdAu_{6.75-x}Al_{0.5+x}$ and $GdAu_{5.3}Al$ (1/1 AC)

S2. Structure refinement details for $GdAu_{6.75-x}Al_{0.5+x}$

S3. Background corrections in heat capacity measurements

S4. Schematic view of the tentative spin configuration

S1. Synthesis details for $GdAu_{6.75-x}Al_{0.5+x}$ and $GdAu_{5.3}Al$ (1/1 AC)

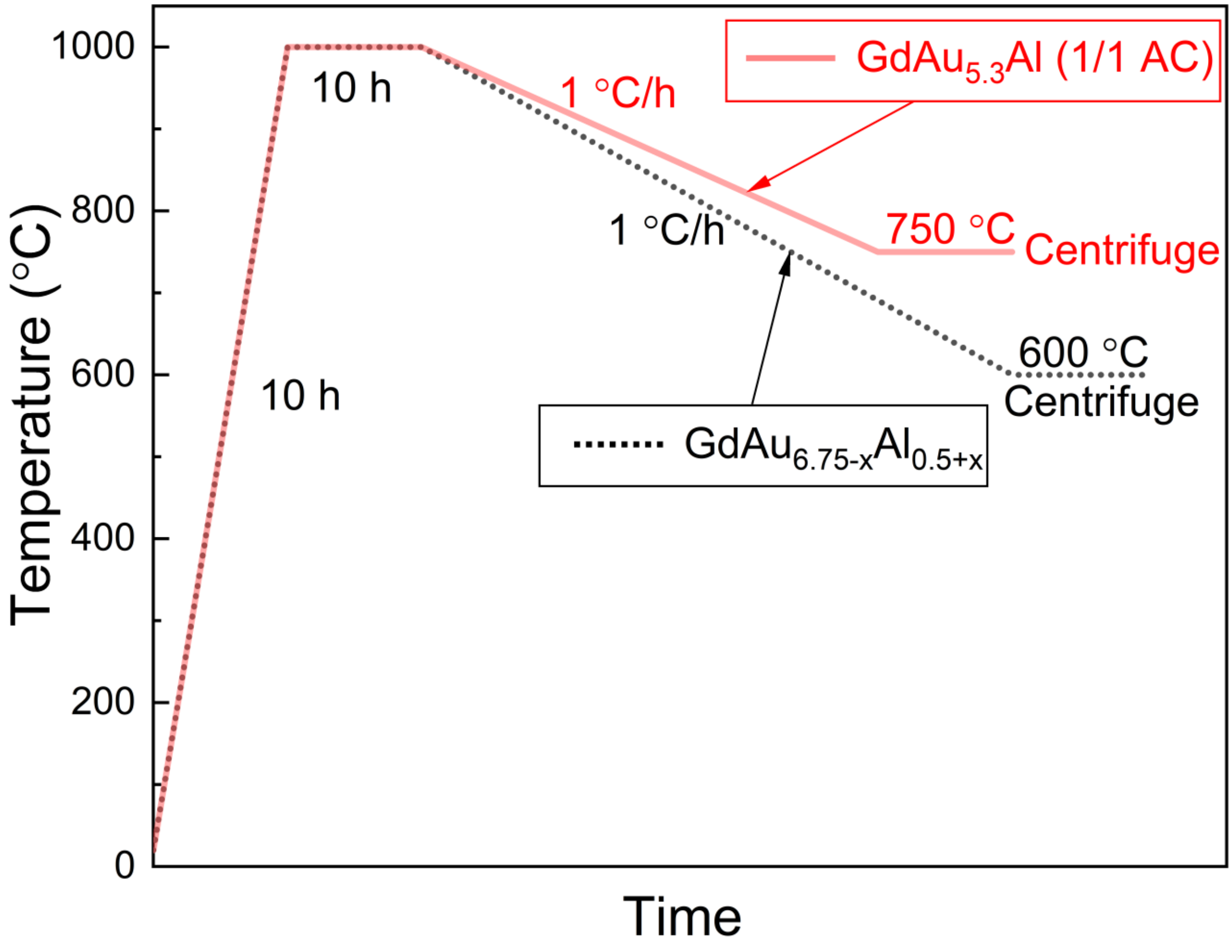

Figure S1: Schematics of temperature protocols used for the synthesis of $GdAu_{6.75-x}Al_{0.5+x}$ and $GdAu_{5.3}Al$ (1/1 AC) samples, starting nominal compositions of $Gd_4(Au_{0.82}Al_{0.18})_{96}$ and $Gd_8(Au_{0.82}Al_{0.18})_{92}$ were used, respectively.

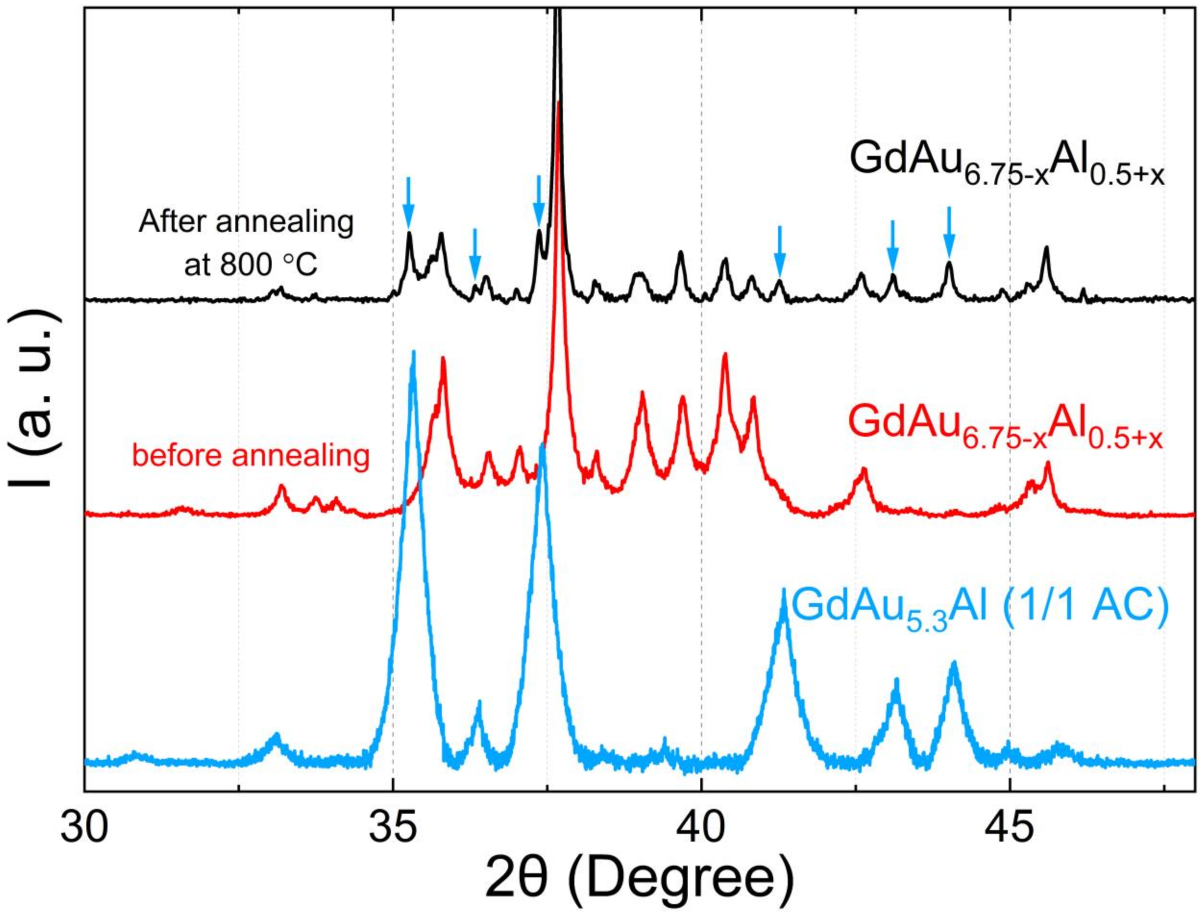

Figure S2: PXRD pattern of the $GdAu_{6.75-x}Al_{0.5+x}$ phase before and after heat treatment. Selected grains from the $GdAu_{6.75-x}Al_{0.5+x}$ sample (~50 mg) were heated to 800 °C at a rate of 10 °C/minute, kept at 800 °C for 1 hour and cooled to room temperature at a rate of 10 °C/minute. The $GdAu_{6.75-x}Al_{0.5+x}$ phase decomposed to $GdAu_{5.3}Al$ (1/1 AC) when heating, and partially reformed during cooling; prominent diffraction peaks from the 1/1 AC phase are indicated by arrows in the annealed sample. The diffraction pattern of the $GdAu_{5.3}Al$ (1/1 AC) is shown for reference. Diffraction background and peaks from Cu-$K_{\alpha2}$ radiation have been subtracted from all patterns for clarity.

S2. Structure refinement details for $GdAu_{6.75-x}Al_{0.5+x}$

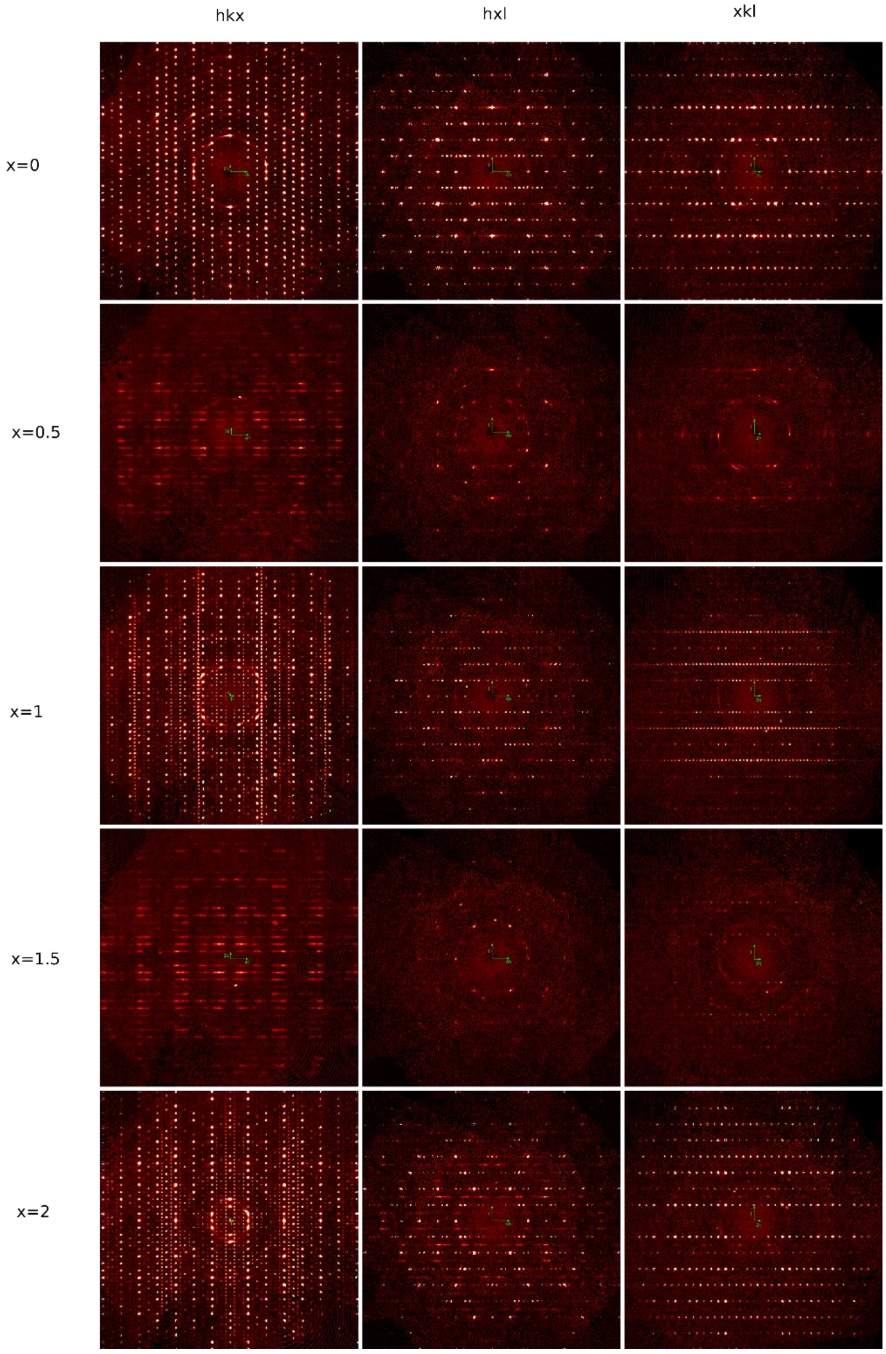

Figure S3: Reciprocal space slices as obtained from SC-XRD data collected for the structure determination of $GdAu_{6.75-x}Al_{0.5+x}$. There are diffuse streaks visible along the h-direction. These streaks originate either from main reflections or appear in between main reflection (e.g. in *hkl* layers with $k = 2m$ and $l = n + 1/2$, where $m,n = 0,1,2,\ldots$) which are signs of both positional and occupational disorder, respectively.

Table S1: Anisotropic atomic displacement parameters for $GdAu_{6.75-x}Al_{0.5+x}$ obtained from SC-XRD refinement.

Atom	U_{11}	U_{22}	U_{33}	U_{12}	U_{13}	U_{23}
Gd1	0.0107(3)	0.0119(3)	0.0104(3)	0.0020(4)	-0.0002(2)	-0.0004(2)
Gd2	0.0100(3)	0.0131(3)	0.0098(3)	0.0014(4)	-0.0011(2)	-0.0002(2)
Al1	0.014(3)	0.012(3)	0.014(3)	0	0.000(2)	0
Al2	0.006(3)	0.009(3)	0.026(4)	0	-0.002(2)	0
Au1	0.0084(3)	0.0117(3)	0.0119(3)	0	-0.0003(2)	0
Au2	0.0083(3)	0.0133(3)	0.0121(3)	0	-0.0007(3)	0
Au3	0.0084(3)	0.0116(3)	0.0135(3)	0	-0.0011(2)	0
Au4	0.0131(3)	0.0159(3)	0.0146(4)	0	0.0063(3)	0
Au5	0.0129(3)	0.0167(4)	0.0144(4)	0	0.0062(3)	0
Au6	0.0124(3)	0.0113(3)	0.0113(3)	-0.0016(3)	0.00062(15)	-0.00036(16)
Au7	0.0123(3)	0.0133(3)	0.0126(3)	0.0019(2)	-0.00009(17)	0.00089(17)
Au8	0.0129(3)	0.0128(3)	0.0104(3)	-0.0002(3)	0.00059(15)	-0.00021(15)
Au9	0.0123(3)	0.0098(3)	0.0112(3)	-0.0003(2)	-0.00142(16)	-0.00088(15)
Au10	0.0129(3)	0.0093(3)	0.0096(3)	0.0020(2)	-0.00032(15)	-0.00101(15)
Au11	0.0117(3)	0.0140(2)	0.0215(3)	-0.0011(3)	0.00225(18)	-0.00170(17)
Au12	0.0133(3)	0.0138(2)	0.0219(3)	0.0013(3)	0.00420(18)	0.00060(17)
Au13	0.0210(4)	0.0144(4)	0.0210(4)	0.0000(2)	-0.0141(3)	-0.0005(2)
Al13	0.0210(4)	0.0144(4)	0.0210(4)	0.0000(2)	-0.0141(3)	-0.0005(2)
Au14	0.0187(5)	0.0148(4)	0.0159(4)	-0.0009(3)	-0.0107(3)	-0.0006(3)
Al14	0.0187(5)	0.0148(4)	0.0159(4)	-0.0009(3)	-0.0107(3)	-0.0006(3)
Au15	0.0197(5)	0.0134(4)	0.0150(4)	0.0013(3)	0.0096(3)	0.0026(3)
Al15	0.0197(5)	0.0134(4)	0.0150(4)	0.0013(3)	0.0096(3)	0.0026(3)
Au16	0.0126(5)	0.0166(6)	0.0105(5)	0.0005(3)	0.0029(3)	0.0010(3)
Al16	0.0126(5)	0.0166(6)	0.0105(5)	0.0005(3)	0.0029(3)	0.0010(3)

Table S2: Complete list of interatomic distance between all atoms up to 5 Å range for $GdAu_{6.75-x}Al_{0.5+x}$ obtained from SC-XRD refinement.

**Atom pair**			**d/Å**	**Atom pair**			**d/Å**
Gd1	Au7	1x	3.1461(13)	Au6	Au15|Al15	1x	2.8373(12)
	Au10	1x	3.1496(13)		Au14|Al14	1x	2.8539(11)
	Au6	1x	3.1505(10)		Au11	1x	2.878(1)
	Au1	1x	3.1514(9)		Au10	1x	2.9711(8)
	Au3	1x	3.1561(9)		Au10	1x	3.0783(8)
	Au9	1x	3.1579(10)		Au9	1x	3.3151(10)
	Au6	1x	3.1818(10)		Au6	1x	4.3266(11)
	Au2	1x	3.1952(8)		Au14|Al14	1x	4.6924(10)
	Au9	1x	3.1977(10)		Au12	1x	4.6993(10)
	Au11	1x	3.1980(9)		Au15|Al15	1x	4.7224(10)
	Au8	1x	3.2262(9)		Au12	1x	4.728(1)
	Au11	1x	3.2304(9)		Au13|Al13	1x	4.8616(10)
	Au13|Al13	1x	3.2767(12)		Au16|Al16	1x	4.8641(13)
	Au14|Al14	1x	3.2780(12)		Au8	1x	4.8800(9)
	Au15|Al15	1x	3.2967(13)		Au8	1x	4.8927(10)
	Au16|Al16	1x	3.3089(14)		Au8	1x	4.8981(9)
	Al1	1x	4.105(5)	Au7	Au16|Al16	1x	2.8467(14)
	Al2	1x	4.131(5)		Au13|Al13	1x	2.8919(11)
	Au2	1x	4.4995(9)		Au9	1x	2.8961(8)
	Au14|Al14	1x	4.9773(11)		Au12	1x	2.8969(10)
	Au15|Al15	1x	4.9916(11)		Au9	1x	3.1273(8)
	Au16|Al16	1x	4.9951(13)		Au10	1x	3.1948(10)
	Au13|Al13	1x	4.9977(11)		Au7	1x	4.3343(11)
Gd2	Au10	1x	3.0941(10)		Au13|Al13	1x	4.6329(10)
	Au9	1x	3.0963(13)		Au11	1x	4.6346(10)
	Au7	1x	3.1031(10)		Au11	1x	4.7778(10)
	Au6	1x	3.1130(13)		Au14|Al14	1x	4.8025(11)
	Au4	1x	3.1259(8)		Au16|Al16	1x	4.8214(12)
	Au5	1x	3.1274(8)		Au15|Al15	1x	4.8235(11)
	Au8	1x	3.1801(9)		Au8	1x	4.8560(9)

	Au12	1x	3.1847(9)		Au8	1x	4.8772(10)
	Au15|Al15	1x	3.1990(13)		Au8	1x	4.9632(9)
	Au12	1x	3.2051(9)	Au8	Au12	1x	2.9585(7)
	Au10	1x	3.2190(9)		Au11	1x	2.9857(7)
	Au7	1x	3.2195(10)		Au14|Al14	1x	2.9957(11)
	Au13|Al13	1x	3.2210(12)		Au16|Al16	1x	2.9970(13)
	Au14|Al14	1x	3.2559(12)		Au13|Al13	1x	3.0123(11)
	Au16|Al16	1x	3.2752(14)		Au11	1x	3.0168(7)
	Al1	1x	3.615(4)		Au16|Al16	1x	3.0172(13)
	Al2	1x	3.620(4)		Au15|Al15	1x	3.0290(12)
	Au3	1x	4.6813(11)		Au15|Al15	1x	3.0319(12)
	Au1	1x	4.7110(11)		Au12	1x	3.0331(7)
	Au14|Al14	1x	4.9330(11)		Au13|Al13	1x	3.0367(11)
	Au4	1x	4.9356(9)		Au14|Al14	1x	3.0556(11)
	Au5	1x	4.9374(9)		Au16|Al16	1x	4.7919(12)
	Au16|Al16	1x	4.9418(13)		Au14|Al14	1x	4.7984(10)
	Au15|Al15	1x	4.9623(11)		Au16|Al16	1x	4.8077(12)
	Au13|Al13	1x	4.9638(11)		Au13|Al13	1x	4.8135(10)
Al1	Au4	1x	2.574(7)		Au15|Al15	1x	4.8167(10)
	Au6	2x	2.630(4)		Au15|Al15	1x	4.8236(10)
	Au10	2x	2.668(4)		Au13|Al13	1x	4.8308(10)
	Au2	1x	2.677(7)		Au14|Al14	1x	4.836(1)
	Au1	1x	2.752(7)		Au9	1x	4.8625(10)
	Au5	1x	2.763(7)		Au10	1x	4.8782(10)
	Au1	1x	3.164(7)		Au9	1x	4.8839(9)
	Au5	1x	3.297(7)		Au12	1x	4.8846(12)
	Au10	2x	4.390(6)		Au12	1x	4.8852(12)
	Au7	2x	4.529(6)		Au10	1x	4.8936(8)
	Al2	1x	4.577(10)		Au11	1x	4.9049(12)
	Au15|Al15	2x	4.5923(12)		Au9	1x	4.9075(9)
	Au3	1x	4.718(7)		Au11	1x	4.9310(12)
	Au6	2x	4.849(6)		Au10	1x	4.9416(8)
	Au9	2x	4.974(6)	Au9	Au13|Al13	1x	2.8505(11)

	Au3	1x	4.983(7)		Au16|Al16	1x	2.8578(14)
Al2	Au5	1x	2.580(6)		Au11	1x	2.8822(10)
	Au9	2x	2.606(4)		Au9	1x	4.3371(11)
	Au7	2x	2.662(4)		Au12	1x	4.6494(10)
	Au2	1x	2.688(6)		Au13|Al13	1x	4.6619(10)
	Au3	1x	2.782(7)		Au12	1x	4.7333(10)
	Au4	1x	2.785(7)		Au16|Al16	1x	4.7453(12)
	Au3	1x	3.126(7)		Au14|Al14	1x	4.8650(11)
	Au4	1x	3.282(7)		Au15|Al15	1x	4.8767(11)
	Au7	2x	4.404(6)	Au10	Au15|Al15	1x	2.8317(12)
	Au10	2x	4.510(6)		Au14|Al14	1x	2.8900(11)
	Au16|Al16	2x	4.6448(14)		Au12	1x	2.9036(10)
	Au1	1x	4.743(7)		Au10	1x	4.3238(10)
	Au9	2x	4.897(7)		Au11	1x	4.6819(10)
	Au1	1x	4.941(7)		Au14|Al14	1x	4.683(1)
	Au6	2x	4.950(6)		Au15|Al15	1x	4.7451(10)
Au1	Au3	1x	2.7476(10)		Au11	1x	4.7517(10)
	Au5	1x	2.8213(11)		Au13|Al13	1x	4.7924(10)
	Au6	2x	2.8256(8)		Au16|Al16	1x	4.8488(13)
	Au2	1x	2.9153(11)	Au11	Au16|Al16	1x	2.6777(13)
	Au10	2x	2.9210(9)		Au14|Al14	1x	2.6951(11)
	Au2	1x	3.0458(11)		Au15|Al15	1x	2.7074(12)
	Au6	2x	4.1284(9)		Au13|Al13	1x	2.7128(11)
	Au9	2x	4.1416(9)		Au12	1x	2.8042(9)
	Au4	1x	4.4647(11)		Au16|Al16	1x	3.8472(13)
	Au14|Al14	2x	4.7096(10)		Au13|Al13	1x	3.8883(11)
	Au4	1x	4.9524(11)		Au15|Al15	1x	3.9024(12)
Au2	Au6	2x	2.8710(9)		Au14|Al14	1x	3.9037(11)
	Au9	2x	2.8892(9)		Au13|Al13	1x	4.5838(10)
	Au3	1x	2.9155(11)		Au14|Al14	1x	4.5964(10)
	Au3	1x	3.0658(11)		Au16|Al16	1x	4.6343(12)
	Au7	2x	4.1055(10)		Au15|Al15	1x	4.6466(11)
	Au10	2x	4.129(1)	Au12	Au16|Al16	1x	2.6760(13)

Au11	2x	4.6153(6)	Au14|Al14	1x	2.6938(11)
Au5	1x	4.6767(11)	Au15|Al15	1x	2.7054(12)
Au4	1x	4.7288(11)	Au13|Al13	1x	2.7081(11)

S3. Background corrections in heat capacity measurements

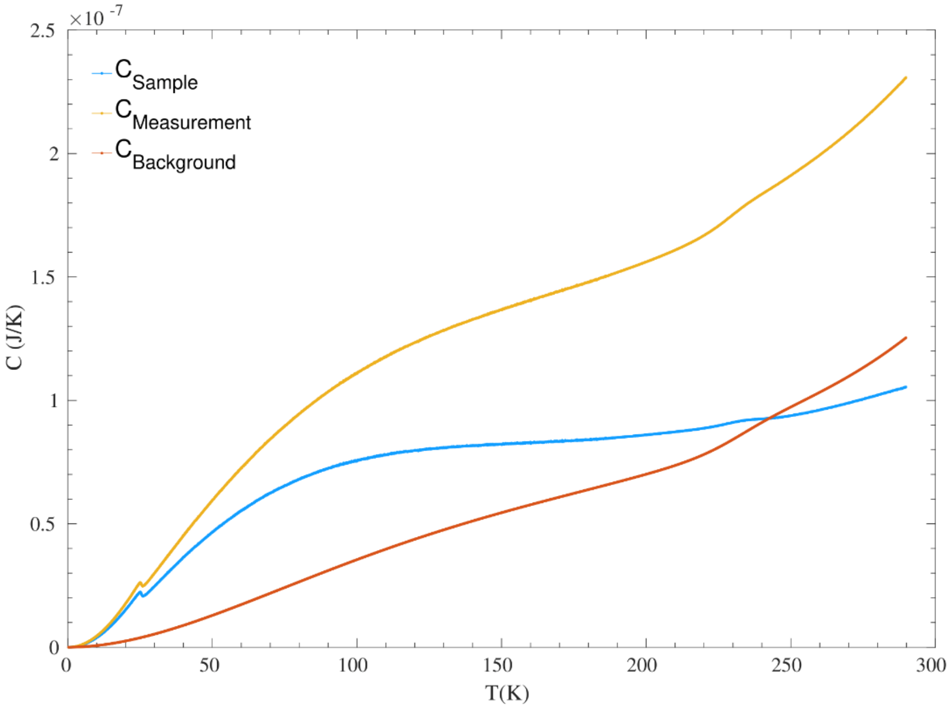

Figure S4: Temperature dependence of Specific heat C(T) of $GdAu_{6.75-x}Al_{0.5+x}$ and the background from the calorimeter membrane and Apiezon grease (recorded under zero magnetic field): Raw data for the measurement of $GdAu_{6.75-x}Al_{0.5+x}$ ($C_{Measurement}$), background data, measured separately and fitted by a function ($C_{Background}$), and the resulting data for $GdAu_{6.75-x}Al_{0.5+x}$ after subtraction of the background data from the raw data (C_{Sample}). The anomaly near 230K is attributed to the Apiezon N-Grease used in the experiments [See *"The specific heat of apiezon N grease", by J. G. Bunting, T. Ashworth, and H. Steeple, Cryogenics* ***9****, 385 (1969)]*, whose contribution is not perfectly compensated by the background subtraction.

S4. Schematic view of the tentative spin configuration

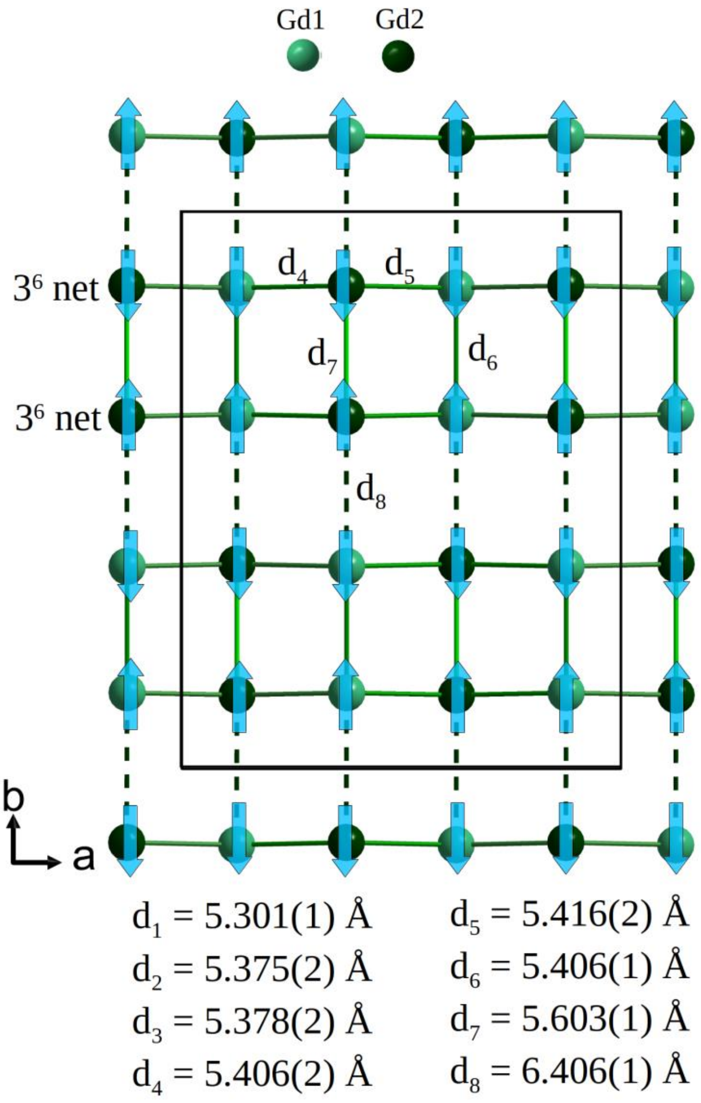

Figure S5. The spin configuration suggested by the magnetometry data is sketched by adding to Figure 4c arrows representing the magnetic moments. The *ac* planes are ferromagnetic, i.e. with all magnetic moments parallel to each other; the *ac* planes are antiferromagnetically coupled along the *b* direction.
